# Temporal dynamics of antimicrobial resistance gene abundances in chicken manure and anaerobic digestate

**DOI:** 10.3389/frabi.2025.1612886

**Published:** 2025-06-27

**Authors:** Aleksandra Atanasova, Thomas Amon, Uwe Roesler, Anika Friese, Roswitha Merle, Tina Kabelitz

**Affiliations:** ^1^ Department Sensors and Modelling, Leibniz Institute for Agricultural Engineering and Bioeconomy e. V. (ATB), Potsdam, Germany; ^2^ Institute for Animal Hygiene and Environmental Health, Veterinary Centre for Resistance Research, Freie Universität Berlin (FU), Berlin, Germany; ^3^ Institute of Veterinary Epidemiology and Biostatistics, Veterinary Centre for Resistance Research, Freie Universität Berlin (FU), Berlin, Germany

**Keywords:** antibiotics, anaerobic digestion fermentation, antimicrobial resistance gene (ARG), poultry, broiler, litter, high throughput qPCR (HT-qPCR)

## Abstract

**Introduction:**

Antimicrobial resistance (AMR) can spread in microorganisms through the transfer of antimicrobial resistance genes (ARGs). Livestock husbandry is one of the pathways for AMR emergence and transmission. Chicken manure contains valuable nutrients for agricultural field fertilization and can be used as input material for biogas production by anaerobic digestion (AD). However, usually, chicken manure also contains quite high levels of ARGs. In this study, we investigated the presence and temporal dynamics of ARGs against different antibiotic classes in chicken manure and anaerobic digestate as a source of AMR spread.

**Methods:**

To get an overview of the ARG profiles, we quantified the abundances of 374 ARGs by high-throughput (HT)-PCR. We studied eight selected ARGs (*tetA*, *tetX*, *sul1*, *sul2*, *lnuF*, *emrD*, *aadA*, and *tnpA*) using qPCR in chicken manure from different flocks and animal ages and in digestate from different AD time points.

**Results:**

Chicken manure showed higher amounts of ARGs compared to digestate, which was characterized by a higher ARG diversity. We observed that the effect of chicken age differed between the flocks. ARG abundances in digestate from different time points and different treatment conditions did not exhibit major changes.

**Conclusion:**

The flocks’ variability had no relevant effect on ARG abundances in chicken manure, likely due to similar growth conditions. However, manure ARG content increased with the age of the chickens. In our experimental batch setup, AD was more effective in reducing AMR microorganisms than reducing ARGs. Further investigations on process optimization or the application of pre-treatment methods could enhance ARG reduction. Notably, pre-mixing chicken manure with material from a biogas plant prior to processing resulted in a lower ARG load compared to untreated chicken manure.

## 1 Introduction

Antibiotic resistance is a global problem [World Health Organization, 2023]. The WHO estimated that 1.27 million deaths were directly attributed to antibiotic resistance and 4.95 million deaths were associated with antibiotic resistance in 2019 worldwide (World Health Organization, 2023). The reason for antibiotic resistance is antimicrobial-resistant (AMR) bacteria harboring antibiotic resistance genes (ARGs), which can spread between bacteria of animals, humans, and the environment (Zhuang et al., 2021). Ding et al. (2023); Skandalis et al. (2021), and Zhuang et al. (2021) described the pathways through which ARGs are transferred between different environments, including hospitals and farms. The connection among all these environments contributes to the One Health concept, which emphasizes the interdependence of human, animal, and environmental health. German livestock husbandry consumed 309 tons of antibiotics in 2023, and, therefore, farms are one of the sources of AMR emergence and spread (German Federal Institute for Risk Assessment, 2025). In 2023, the European Union (EU) produced 20.6 tons of pork and 13.3 tons of poultry meat (eurostat Statistics Explained, 2024), leading to huge manure production. Köninger et al. (2021) reported that annually approximately 1.4 billion tons of livestock manure were produced in the EU and the United Kingdom between 2016 and 2019. Manure is a valuable source for agricultural field fertilization as it contains different nutrients. However, it can transfer different pollutants, such as ARGs, into the environment (Peng et al., 2022). One suitable way to use manure for energy (biogas) production and pollution reduction is anaerobic digestion (AD) (Congilosi and Aga, 2021; Zhang et al., 2013, 2012). This process is suitable for the reduction of pathogens and AMR bacteria (Atanasova et al., 2025; Thomas, 2023). AD also leads to ARG reduction (Mortezaei et al., 2024; Song et al., 2017).

The following genes are examples of those that are widespread and of particular concern due to their relevance in both human and animal health. The genes *emrD* and *tetA* encode efflux pumps. The genes *tetX* and *lnuF* encode enzymes that inactivate antibiotic molecules. Resistance to aminoglycosides can be transferred by aminoglycoside nucleotidyltransferase encoded by *aadA*. The genes *sul1* and *sul2* encode an enzyme that replaces the antibiotic target in the bacterial cell, therefore, enabling resistance to sulfonamide (CARD: https://card.mcmaster.ca/analyze/rgi; Alcock et al., 2023). Together with ARGs, mobile genetic elements (MGEs) play a role in transferring antibiotic resistance. One example that is frequently encountered in the environment is the transposon *tnpA*, which was shown to be associated with antibiotic resistance (Partridge et al., 2018). Associations between MGEs and ARGs could indicate horizontal gene transfer (Waseem et al., 2019). Investigating ARGs and their dynamics in chicken manure and during AD helps to assess AMR spread in the environment. There are only a limited number of studies that compare the amount of frequent ARGs in chicken manure from different flocks, animal ages, and at different AD time points. Therefore, the aims of this study were 1) to identify frequently appearing ARGs in chicken manure and anaerobic digestate from chicken manure, 2) to investigate the variation in ARG abundances in chicken manure from different flocks and chickens of different ages, and 3) to determine ARG temporal dynamics during AD in the mesophilic temperature range.

## 2 Materials and methods

### 2.1 Study design and sampling

Details of the performed AD experiments were described in the study of Atanasova et al. (2025). In brief, chicken manure for AD was collected from a conventional broiler farm in Brandenburg, Germany. Inoculum for AD was collected from a biogas plant running on maize silage and cow slurry. Four different setups were used for the experiment: 1) AD with chicken manure at 30°C, 2) AD with chicken manure and sawdust addition at 30°C, 3) AD with chicken manure at 37°C, and 4) AD with chicken manure and sawdust addition at 37°C. Sawdust additions served as an additional carbon source and optimized the AD reaction to a higher biogas yield. During the AD experiment, digestate samples were collected every day from day 0 until day 9 and every second day from day 10 until day 20 and stored at -20°C. For ARG quantifications by qPCR, we selected digestate samples from days 0, 1, 3, 6, 14, 17, and 20 (**Table 1**). Samples from day 0 and day 1 were chosen as representatives of the starting condition at the beginning of the AD experiment. Microbiological cultivation results (Atanasova et al., 2025) showed a rapid change in *E. coli* survival during the first week of the experiment. Therefore, we performed qPCR analyses of samples from day 3 as a middle time point before *E. coli* was undetectable. On day 6, cultivated *E. coli* were under the detection limit at 37°C. On day 14, *E. coli* in digestate samples from reactors at 30°C were below the detection limit (see Atanasova et al., 2025). Day 17 was the last day of microbiological and chemical analyses and day 20 was the last day of sampling.

**Table 1:** Digestate samples.

Sample	Time, day	Temp, °C	Content of digestate
1	0	30	Manure
2	0	30	Manure with sawdust
3	0	37	Manure
4	0	37	Manure with sawdust
5	1	30	Manure
6	1	37	Manure
7	3	30	Manure
8	3	37	Manure
9	6	30	Manure
10	6	30	Manure with sawdust
11	6	37	Manure
12	6	37	Manure with sawdust
13	14	30	Manure
14	14	30	Manure with sawdust
15	14	37	Manure
16	14	37	Manure with sawdust
17	17	37	Manure
18	17	30	Manure
19	17	37	Manure with sawdust
20	20	37	Manure
21	20	30	Manure with sawdust
22	20	37	Manure with sawdust

For monitoring selected ARGs in chicken manure, broiler litter was collected from the same conventional farm in Brandenburg, Germany a few months later. We took samples from five different flocks (4, 5, 6, 10, and 11) at three different time points (chickens that were 1 week, 3 weeks, and 5 weeks old). Additionally, the farmer provided us with information regarding the medical treatments (date, drug, dose, and duration) for each flock.

### 2.2 DNA isolation

DNA isolation from chicken manure was performed using a QIAamp Fast DNA Stool Mini Kit (QIAGEN, Hilden, Germany) according to the manufacturer’s instructions. For DNA isolation from the digestate samples, the samples were processed according to the manufacturer’s instructions using the Soil FastDNA™ SPIN Kit (MP Biomedicals, California, USA). DNA concentration and purity were measured using a NanoDrop (NanoPhotometer^®^ N60/N50, IMPLEN, Munich, Germany).

### 2.3 HT-qPCR SmartChip

To get an overview of the ARG composition and abundance in chicken manure and digestate, we sent one mixture of chicken manure DNA and one mixture of anaerobic digestate DNA to Resistomap (Helsinki, Finland). We pooled several DNA samples from different time points together to cover a wider range of the presented ARGs. In the results, we named the samples “Chicken manure” that contained DNA of 1-week and 3-week-old chicken manure, from 2 and 3 different barns, respectively. “Digestate” samples consisted of DNA samples from AD with chicken manure at 37°C on day 3 and 14; chicken manure and sawdust at 37°C on day 6, and at 30°C on day 14. At Resistomap, a SmartChip HT-qPCR (Wafergen Inc, USA) with 384 genes (374 ARGs, 9 taxonomic genes, and 16S rRNA) was performed. The abundance of each gene was quantified in triplicate. As a result, relative abundances were calculated using the ΔC method, with 16S rRNA serving as the reference gene. The selection of ARGs was the standard setup of the ARG2.1 chip or the One Health Package. ARGs measured by the chip represented resistances against all the important antibiotic classes, some integrons, and MGEs associated with AMR. A detailed list of ARGs measured by HT-qPCR is shown in **Supplementary Figures 1**, **2**. As output, we obtained gene abundances relative to 16S rRNA and gene copy numbers.

### 2.4 qPCR

We investigated the detailed abundance and temporal dynamics of eight ARGs (*aadA*, *emrD*, *lnuF*, *tetA*, *tetX*, *sul1*, *sul2*, and *tnpA)* in chicken manure and digestate samples using qPCR. One of the main criteria for ARG selection was their high abundance detected by the SmartChip system, reflecting their widespread occurrence in the environment. Additional selection criteria for qPCR genes included the use of specific antibiotic classes on the farm during the study period, such as sulfamethoxazole and lincomycin, and the clinical relevance of these ARGs in both human and veterinary medicine. Primer (Merck Millipore, Missouri USA) information is shown in **Table 2**. The efficiency of all the primers was tested and it was 90%–100%. All samples were measured in triplicate. Each reaction mixture contained 20 µL, including 1 µL of DNA, 10µL SYBR Green Master I (Light Cycler 480 SYBR Green I Master, Roche), 1 µL forward primer, 1µl reverse primer (10 mM), and 7 µL MiliQ water. Samples were measured in a thermocycler (BioRad CFX384 Real-Time system, California, USA) under the following program: pre-incubation cycle for 180s at 95°C, 40 cycles of amplification for 10s at 95°C, primer annealing for 20 s at the primer-specific temperature (55°C-62°C, according to **Table 2**), and elongation for 20 s at 72°C. Additionally, a melting curve analysis was performed from 65°C to 95°C with a temperature increase of 0.5°C every 5s. The abundances of all the ARGs were normalized to 16S rRNA abundance. Fold change values were calculated by dividing the abundance of the measured samples by the reference sample, which was always the first one in the time series (week 1 for chicken manure and day 0 for AD).

**Table 2:** Primer list.

Name	Forward (5’-3’)	Reverse (5’-3’)	Reference	Tm, °C
*aadA*	GTTGTGCACGACGACATCATT	GGCTCGAAGATACCTGCAAGAA	Muurinen et al. (2017)	60
*emrD*	CTCAGCAGTATGGTGGTAAGCATT	ACCAGGCGCCGAAGAAC	Okonkwo (2023)	60
*InuF*	ATACCGGTCATTTCCACTTGGC	GCATCAGGCTGATGAGGTTCAA	Muurinen et al. (2017)	62
*sul1*	GCCGATGAGATCAGACGTATTG	CGCATAGCGCTGGGTTTC	Mortezaei et al. (2024)	59
*sul2*	GATATTCGCGGTTTTCCAGA	CGCAATGTGATCCATGATGT	Jaleta et al. (2024)	55
*tetA*	CTCACCAGCCTGACCTCGAT	CACGTTGTTATAGAAGCCGCATAG	Mortezaei et al. (2024)	60
*tetX*	AAATTTGTTACCGACACGGAAGTT	CATAGCTGAAAAAATCCAGGACAGTT	Stedtfeld et al. (2018)	60
*tnpA*	CCGATCACGGAAAGCTCAAG	GGCTCGCATGACTTCGAATC	Mortezaei et al. (2024)	58
16S rRNA	GGGTTGCGCTCGTTGC	ATGGYTGTCGTCAGCTCGTG	Mortezaei et al. (2024)	59

### 2.5 Statistical analysis

Statistical analysis was performed using IBM SPSS Statistics, version 25 (SPSS, Inc., Chicago, IL). We applied a linear regression analysis with the fold change of genes as the dependent variable and several independent variables [for AD: days, temperature, and substrate additive (sawdust); chicken manure: time and flock]. All two-way interactions were included, and non-significant ones were removed stepwise. Hochberg’s *post-hoc* tests were run for pairwise comparisons of the days (AD) or time and flock (chicken manure). Confidence intervals at 95% were calculated when applicable. A *p*-value <0.05 was considered significant.

## 3 Results

### 3.1 ARGs by HT-qPCR in chicken manure and digestate

**Figure 1:**
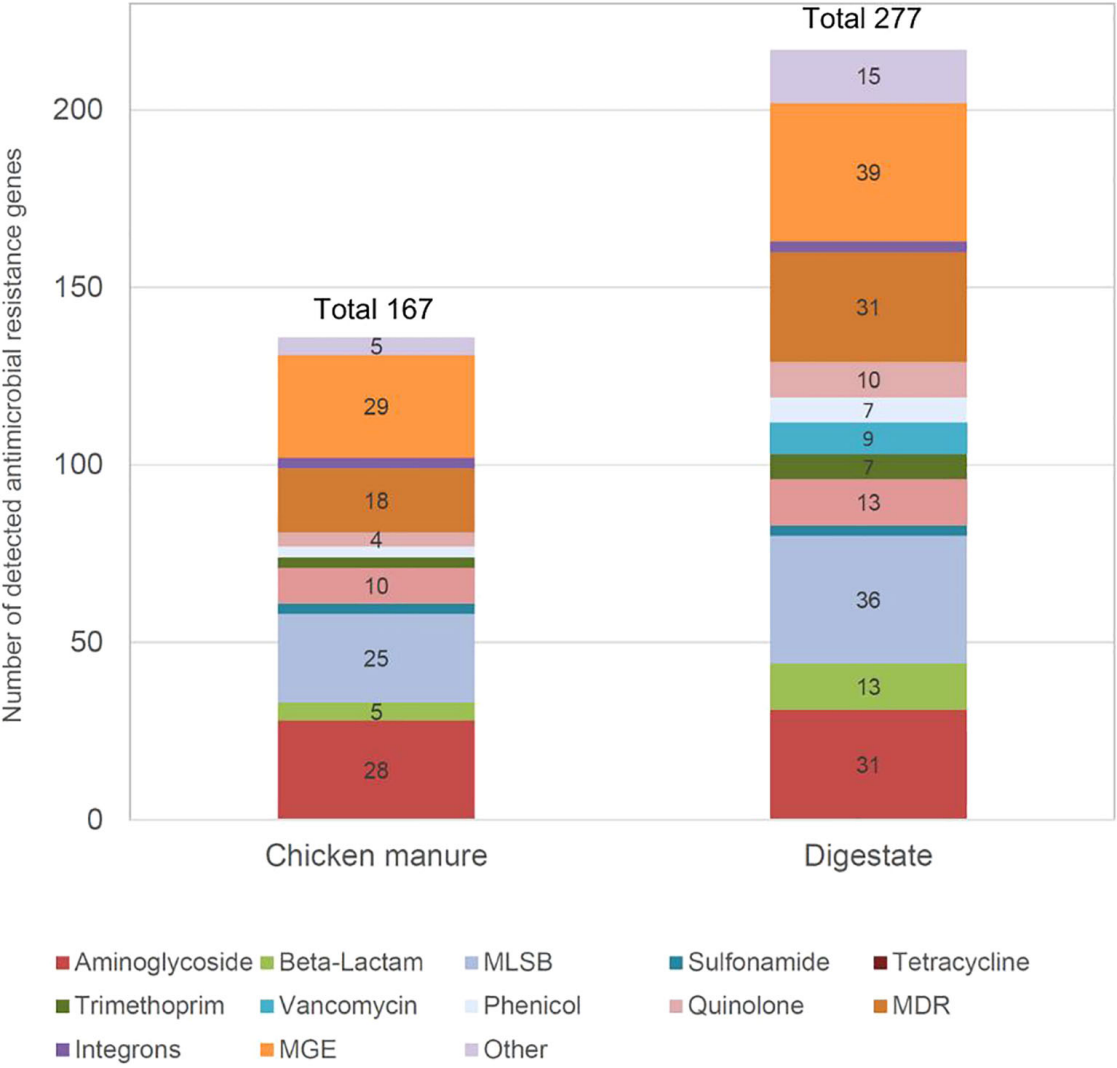
Number of detected antimicrobial resistance genes using a Resistomap HT-qPCR SmartChip in chicken manure and digestate. MDR, multidrug resistance; MLSB, macrolide-lincosamide-streptogramin B; MGEs, mobile genetic elements.

Out of 374 ARGs potentially detectable on the Resistomap ARG2.1 chip, the maximum detected ARG number was 277 in digestate compared to 167 in chicken manure (**Figure 1**). In the digestate sample, the number of different detected ARGs was 110 (approx. 66%) more than in the chicken manure sample. This meant that the ARG diversity in the digestate was higher than in chicken manure. The ARG classes with the most detected genes were in both types of samples: MGE, multidrug resistance (MDR), aminoglycosides, and macrolide-lincosamide-streptogramin B (MLSB). In chicken manure, the two dominant ARG classes were MGE (29 detected genes) and aminoglycosides (28 detected genes), whereas for digestate, the two dominant ARG classes were MGE (39 detected genes) and MLSB (36 detected genes).

**Figure 2:**
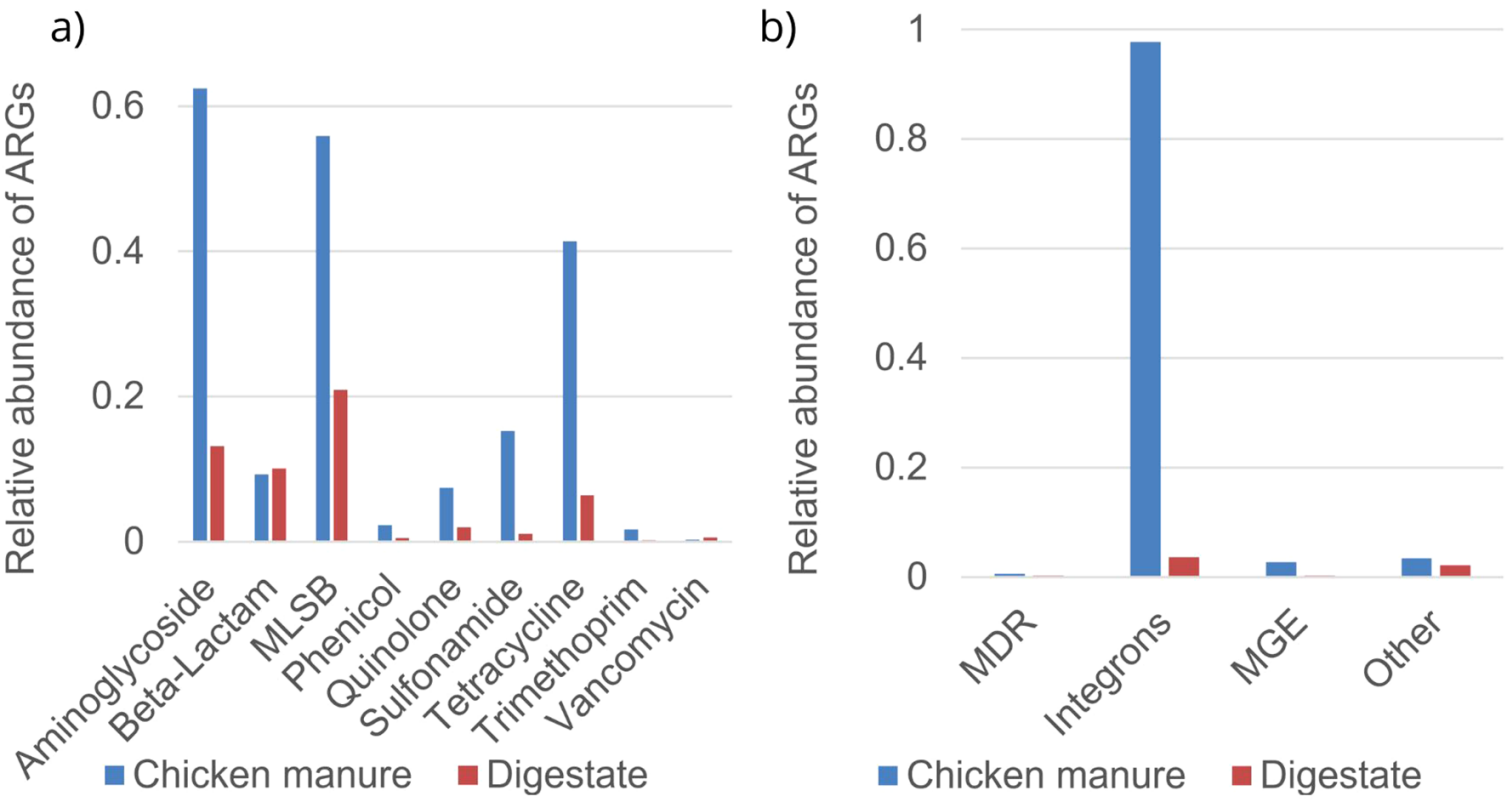
Average relative abundance normalized to 16S rRNA of antibiotic resistance gene (ARG) classes using a Resistomap HT-qPCR SmartChip in chicken manure and digestate. **(a)**: according to antibiotic class, **(b)** according to multidrug resistance (MDR), integrons, mobile genetic elements (MGEs), and others.

The relative abundances of ARG classes in chicken manure were much higher compared to digestate (**Figure 2**), except for beta-lactams, where both were similar (relative abundance in chicken manure was 0.093 and in digestate was 0.101). The highest relative abundance was observed for integrons class, consisting of four different genes (**Supplementary Figures 1**, **2**), with 0.978 for chicken manure and 0.036 in digestate. The ARGs with the highest relative abundance in chicken manure were the aminoglycoside, MLSB, and tetracycline classes. Similarly, digestate showed the highest abundances of the aminoglycoside, MLSB, and beta-lactam ARG classes.

The analyses of the relative abundances of single ARGs (**Supplementary Figures 1**, **2**) in the aminoglycoside group revealed that the genes *aadA_1* (0.079), *aadA1_2* (0.071), and *aadA2_3* (0.069) showed high detection frequencies in chicken manure, while *aadA7* (0.028) was predominantly in digestate. In chicken manure, the MLSB class gene *lnuC* (0.018) was the most abundant, while in digestate, the dominant genes were *lnuB* (0.037) and *ermF_1* (0.035). Chicken manure contained high levels of *sul1* (0.004) and *sul2* (0.012) from the sulfonamide group, as well as *tet39* (0.246), *tetX* (0.098), and *tetW* (0.079) from the tetracycline class. Tetracycline resistance genes *tetM* (0.011) and *tetD* (0.008) were mainly present in the digestate. In the integrons class, *intI1_1* (1.73) and *intI2_2* (0.083) were highly abundant in chicken manure and *intI3* (0.032) was more frequent in digestate. Chicken manure showed a high abundance of the MGE, *tnpA_1* (0.120), while digestate had high levels of *trbC* (0.012).

### 3.2 ARGs in chicken manure by qPCR

After getting an overview of the ARG diversity and abundances in chicken manure and the anaerobic digestate by the SmartChip HT-qPCR, we focused on a more detailed investigation of eight selected ARGs using qPCR. All the selected genes (*tetA*, *tetX*, *sul1*, *sul2*, *tnpA*, *emrD*, *InuF*, and *aadA*) were detectable in chicken manure from animals of different ages and from different flocks.

**Figure 3:**
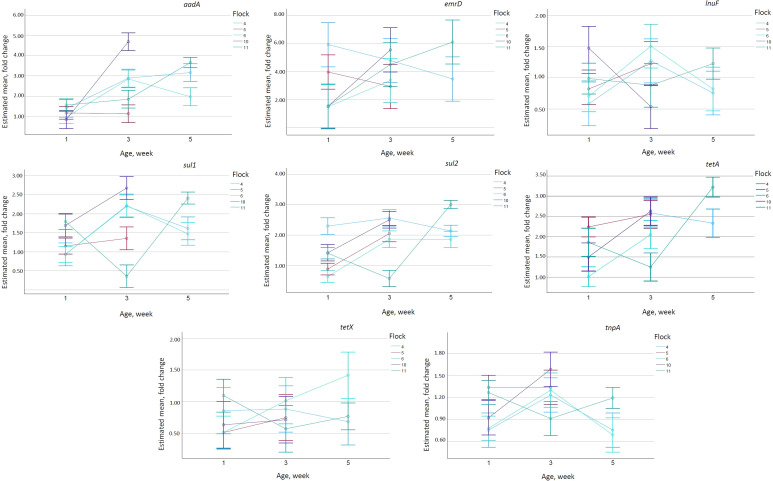
Estimated mean from UNIANOVA model for all of selected genes (*aadA*, *emrD*, *lnuF*, *sul1*, *sul2*, *tetA*, *tetX*, and *tnpA*). Non-estimated means are not plotted. Error bars show 95% confidence interval.

Age and flock significantly influenced all the genes, but the effect of age differed between the flocks (**Supplementary Table 1**). The abundance of the *tetA* gene increased between week 1 and week 3, except for flock 4, where it decreased and had a maximum on week 5. Flock 6 had significantly lower values (*p <*0.001) than all other flocks (**Figure 3**). Concerning *tetX*, the values increased significantly in flock 6 (*p <*0.001), but remained relatively stable in all other flocks. In flocks 4 and 6, *tnpA* had the highest values on week 3, while flock 11 had its lowest values at that time point. Concerning *sul1* and *sul2*, flock 11 again had the lowest values on week 3, with all other flocks peaking on week 3. The abundance of the *emrD* gene had increasing trends in flocks 6, 10, and 11, but decreased in flocks 4 and 5. The values of lnuF peaked on week 3 in flocks 4 and 6, while flock 11 showed the highest values on week 5. Flocks 4 and 6 had very similar abundances of *tnpA*, *sul1*, and *lnuF* (**Figure 3**).

### 3.3 ARGs in digestate by qPCR

The results of the regression models for the digestate samples are shown in **Supplementary Table 2**. For all genes, except for *emrD* and *tetX*, sawdust had no significant impact, while temperature had a significant impact on the fold change of all genes. The genes *aadA*, *tetA*, *sul1*, *sul2*, and *tetX* had significantly lower fold changes at 30°C compared to 37°C, while *emrD* had higher values at 30°C. Regarding the substrates, significant interactions between the substrate and the temperature were observed for *tetX*. For the *emrD* gene, the effect of substrate also differed between the days, with higher values in the sawdust group at days 6, 14, and 20, but not at day 0. Interactions between day and temperature occurred for both *emrD* and *tetX*. These genes had interactions between the independent parameters day-sawdust (*p* = 0.016, resp., 0.015) and day-temperature (*p <*0.001, resp., 0.005), meaning temperature and sawdust were significant depending on the day.

## 4 Discussion

### 4.1 ARGs by HT-qPCR

In our study, ARGs from the aminoglycoside, MLSB, and tetracycline classes were dominant in chicken manure. Similar findings were reported by Peng et al. (2022); Błażejewska et al. (2022), and Sun et al. (2024), who investigated ARGs in manure from broiler farms and confirmed our results. Chicken manure is used for biogas production. Notably, Germany is the largest biomethane producer in Europe (European Biogas Association, 2020). Already in 2018, 9,000 biogas plants were performing anaerobic digestion (Appunn, 2016). The AD process starts with hydrolytic and fermentative bacteria, followed by acetogens and methanogens (Yangin-Gomec et al., 2020), which come from biogas plants, mainly consisting of maize silage and cattle slurry, and finally, chicken manure is mixed with this inoculum. In our digestate (chicken manure and inoculum), the majority of fresh matter by weight (approx. 99%) came from maize silage and cattle slurry. However, this is only 6% of the dry matter weight because the highest proportion of digestate is water. We compared raw material (manure) to digestate (manure with inoculum) to estimate the impact of the inoculum on chicken manure. Due to the fact that our digestate contained a mixture of biogas plant inoculum and chicken manure, it had higher ARG diversity and lower ARG relative abundance (**Figure 2**). Peng et al. (2022) compared the ARG content in different types of manure. Cattle slurry showed the lowest abundance of ARGs compared to poultry and pig manure, which is in agreement with our study. The differences in ARG content can be attributed to variations in the microbiome. Several studies have investigated this question. Peng et al. (2022) and Wang et al. (2019) related ARG abundance to different microbiome compositions in chicken manure and cattle slurry. Yue et al. (2022) and Gurmessa et al. (2021) considered *Firmicutes*, *Proteobacteria*, *Actinobacteria*, and *Bacteroidetes* to be phyla associated with ARG abundance. Gurmessa et al. (2021) reported *Firmicutes* and *Bacteroidetes* were the dominant phyla in cattle slurry, while *Firmicutes* and *Actinobacteria* were the prevalent phyla in poultry manure. Additionally, the microbiome changed during the AD process, which could contribute to ARG changes as well. Riaz et al. (2020) reported a slight increase in the prevalence of the phyla *Firmicutes*, *Proteobacteria*, *Actinobacteria*, and *Bacteroidetes* after anaerobic digestion. This can lead to no decrease or even an increase in ARG abundance. Yangin-Gomec et al. (2020) reported differences between inoculum and digested chicken manure microbiomes. The inoculum, which initially contained a diverse range of phyla without a high prevalence of any specific one, became a digestate with a dominant prevalence of *Firmicutes* and *Actinobacteria*, which are associated with the abundance of ARGs.

The chemical characteristics of poultry manure and cattle slurry contribute to different microbiomes, leading to different ARG abundances, as indicated by Gurmessa et al. (2021). One noticeable difference is the moisture content in cattle slurry (∼ 86%) compared to chicken manure ( ∼ 24%), which is likely to cause microbiome differences. Peng et al. (2022) described a correlation between the chemical characteristics and ARG abundances of cow and sheep manure, which have low ARGs abundance and a poor nutritional content compared to poultry and pig manure, which have a higher amount of ARGs and a richer nutritional content. This suggests that there may be significant differences between raw chicken manure and digestate, which are the result of natural changes in chemical characteristics and microbiome composition during the anaerobic digestion process. Lower ARG relative abundances in digestate might be a sign of AMR reduction, but higher diversity in the future under certain conditions could lead to an increase in AMR.

WHO reported that aminoglycosides are critically important antimicrobials and tetracycline, lincomicide and sulfonamide are highly important antimicrobials. These antibiotics are frequently used in veterinary and human medicine (World Health Organization, 2024). Genes *aadA* (aminoglycosides), *tetA*, *tetX* (Tetracycline), *sul1*, *sul2* (sulfonamides), and *lnuF* (lincomicides) were detected in our study by HT-qPCR. Gene *tetA* encodes an efflux pump for tetracycline residues. Another example of an efflux pump encoding is *emrD*. This pumps efflux of amphipathic molecules (Yin et al., 2006) and belongs to MDR ARGs. Gene *tnpA* is associated with ARGs because it transfers ARGs. Li et al. (2020) reported genes *tetX* and *sul2* on the same plasmid with *tnpA*, and Tada et al. (2016) reported *sul1*. Allmeier et al. (1992) reported transposon Tn1721, including *tnpA* sequence, with gene *tetA*.

### 4.2 ARGs in chicken manure by qPCR

Chicken manure, as a raw material for anaerobic digestion, was investigated, aiming to identify ARG developmental trends in the farms. First, we focused on ARG diversity in chicken manure from different flocks. We analyzed the association of the fold change observations with the flocks’ diseases and treatments. At the beginning of the fattening period, flocks 10 and 11 had colibacillosis and *Enterococcus* infection, while flocks 4, 5, and 6 had colibacillosis and dysbacteriosis. All flocks received the same treatment of lincomycin, sulfamethoxazole and thrimethoprim, corresponding to the genes *lnuF*, *sul1*, *sul2* (selected genes), *dfrA*, and *dfrK*, which had relative abundances similar to *tetA*, *tetR*, and *tetM* (**Supplementary Figure 1**). Burow et al. (2020) correlated AMR with antibiotic usage, including aminoglycosides, sulfamethoxazole, and trimethoprim. The flocks in our study were significantly different from each other, but we did not observe any association or difference between single flocks. Peng et al. (2022) compared the relative abundances of ARGs in broiler manures from different farm sizes and other types of manure. In chicken manure, single genes, such as *tetX*, *cfr*, and *aadA1*, varied between the farms (up to 10^4^), while for *cfxA*, *tet(32)*, and *tetO_02*, the differences in relative abundances were much lower (approximately 10^2^). Peng et al. (2022) reported a significant difference between broilers raised under traditional and intensive conditions. ARG levels were higher in those from intensive farms due to high density and intensive feeding. However, no association between ARG levels and farm type for other livestock species was found. Another factor we considered in our study was age, because manure characteristics are impacted by chicken age (Prado et al., 2022). There was a general trend for all investigated ARG abundances as they all increased with chicken age. During the fattening period, the temperature in the barn is approximately 25°C, and chicken’s body temperature is approximately 40°C. This is a suitable temperature range for the spread of human pathogens (temperature 35°C–37°C) (Gutierrez et al., 2018). An increase in bacteria could cause a spread of AGRs by mobile genetic elements.

### 4.3 ARGs in digestate by qPCR

To sum up the results, even though there have been observed some significant differences between individual days and different AD setups (**Supplementary Figure 3**), the differences were marginal (lower than two-fold) and therefore negligible. Zahedi et al. (2022) reported a significant decrease only of *qnrS* genes, while other genes, including *sul1* do not show changes after AD. Additionally, an increased temperature (55°C compared to 35°C) did not result in a higher ARG reduction. Similarly, Riaz et al. (2020) reported no change in the relative abundance of ARGs such as *sul1*, *aadA*, and *tetX* during the first 5 days of semi-continuous chicken manure AD and mixing with sawdust. Zhang et al. (2019) observed changes in the relative abundance of *tetX* mostly after day 20. In our study, the experiment was stopped after 20 days, the ARG changes that were analyzed during the first 20 days showed no changes in *tetX* fold change. A stable ARG level during the AD process was observed for *tetM*, *emrF*, *mefA*, and *sul2* by Zhang et al. (2019), and in our study as well, except for *emrD*, which slightly increased during AD. ARGs can be transferred across various environments, making them difficult to track. Although several models have been developed to predict their spread, one of the most recent approaches involves the use of machine learning (Li et al., 2018). This complexity contributes to the challenge of accurately predicting and quantifying ARGs that pose a significant risk to human health. Zhang et al. (2019) reported changes in the microbial community during anaerobic digestion. On day 0, the dominant phyla were *Firmicutes* and *Proteobacteria*, and their relative abundances remained stable over 20 days. In contrast, the abundance of *Bacteroidetes* increased by approximately 20%, while *Actinobacteria* remained steady at approximately 5%, making it the fourth-most prevalent phylum. All of these phyla are known to be associated with ARG abundance. These findings suggest that the dominant phyla remained largely unchanged during the digestion process, indicating that ARGs likely had stable microbial hosts to persist and proliferate.

Anjum et al. (2017) showed that after thermophilic AD of poultry manure, selected ARGs were no longer detectable, except for *sul1* and *sul2*. Song et al. (2017) investigated ARGs in swine manure and observed an increase in the abundance of *tetX*, *sul2*, and *tetQ* during the AD process. Song et al. (2017) tested the influence of different biomasses (swine manure mixed with different amounts of wheat straw). The experimental setup with the highest manure content showed maximum abundance of ARGs on day 3, while samples with less manure had their maximum on day 25. Additionally, the experimental setup with a higher proportion of manure to wheat (7:3) showed a decrease in total ARG abundances. Furthermore, Gao et al. (2022) reported a decrease in the abundance of *sul1* and *emrC* in pig manure during AD in a high-solid anaerobic system at a mesophilic temperature, but the abundance of genes *sul2* and *aadA* did not show a strong decrease. In our study, sawdust addition had no relevant impact on ARG loads, similar to the findings of the microbiological analysis (Atanasova et al., 2025), where authors reported no significant differences between *E. coli* concentration with and without sawdust. Based on the studies by Gao et al. (2022) and Song et al. (2017), anaerobic digestion is more suitable for ARG reduction in AD of swine manure compared to poultry manure. AD showed good results in reducing pathogens and AMR bacteria (Atanasova et al., 2025; Thomas, 2023). Furthermore, ARG reduction was achieved through AD of sewage sludge (Mortezaei et al., 2024) and pig manure (Anjum et al., 2017; Song et al., 2017). In our research, AD did not show a reduction in the abundance of ARGs, which remained stable in our setups with chicken manure. However, AMR results obtained by microbiological cultivation (Atanasova et al., 2025) showed a rapid decrease in the number of viable *E. coli* during AD. The selective cultivation method for AMR analyses is labor-intensive, selective of only a limited number of bacterial species, and restricted to viable bacteria. This means that selective cultivation alone is not able to cover the abundance and distribution of ARGs within a sample. qPCR enables the selection of more bacterial species and viability-independent, sensitive, and precise quantification of selected ARG abundances. Therefore, AD chicken manure is a suitable process for the reduction of AMR *E. coli*, while we did not observe a reduction of ARGs in our laboratory batch setups.

## 5 Conclusion

This study has confirmed that ARGs are present in high amounts in chicken manure. The types and levels of ARGs can vary depending on factors such as chicken feeding, keeping, health, treatment, animal age, and microbiome composition. In this study, we demonstrated that the trend of ARG abundances increased with the chickens’ age, while the ARG profiles between different flocks varied only marginally. Chicken manure was mixed with inoculum from a biogas plant that contained cattle slurry and maize silage for anaerobic digestion, which decreased the total ARG levels but increased ARG diversity. While AD is a common and suitable process for pathogen and AMR bacteria reduction, we did not observe a reduction of selected ARGs during the AD process in our laboratory batch experiments. This means that the risk of ARG spread to the environment by manure field fertilization may not be reduced by AD. Even if pathogens and AMR bacteria are reduced after AD, ARGs could be integrated into bacteria from the environment, and hence, acquire and spread antibiotic resistance.

## Data Availability

The original contributions presented in the study are included in the article/**Supplementary Material**. Further inquiries can be directed to the corresponding author.

## References

[B1] Alcock B. P. Huynh W. Chalil R. Smith K. W. Raphenya A. R. Wlodarski M. A.. (2023). CARD 2023: expanded curation, support for machine learning, and resistome prediction at the Comprehensive Antibiotic Resistance Database. Nucleic Acids Res. 51, D690–D699. doi: 10.1093/nar/gkac920 36263822 PMC9825576

[B2] Allmeier H. Cresnar B. Greck M. Schmitt R. (1992). Complete nucleotide sequence of Tn1721: gene organization and a novel gene product with features of a chemotaxis protein. Gene 111, 11–20. doi: 10.1016/0378-1119(92)90597-I 1312499

[B3] Anjum R. Grohmann E. Krakat N. (2017). Anaerobic digestion of nitrogen rich poultry manure: Impact of thermophilic biogas process on metal release and microbial resistances. Chemosphere 168, 1637–1647. doi: 10.1016/j.chemosphere.2016.11.132 27932039

[B4] Appunn K. (2016). Bioenergy - the troubled pillar of the Energiewende, Biogas in Germany’s energy transition.

[B5] Atanasova A. Amon T. Friese A. Rösler U. Merle R. Herrmann C.. (2025). Effects of carbon–to–nitrogen ratio and temperature on the survival of antibiotic-resistant and non-resistant escherichia coli during chicken manure anaerobic digestion. Poultry 4. doi: 10.3390/poultry4010009

[B6] Błażejewska A. Zalewska M. Grudniak A. Popowska M. (2022). A comprehensive study of the microbiome, resistome, and physical and chemical characteristics of chicken waste from intensive farms. Biomolecules 12. doi: 10.3390/biom12081132 PMC9406075 36009027

[B7] Burow E. Grobbel M. Tenhagen B.-A. Simoneit C. Szabó I. Wendt D.. (2020). Antibiotic resistance in escherichia coli from broiler chickens after amoxicillin treatment in an experimental environment. Microb. Drug Resist. 26, 1098–1107. doi: 10.1089/mdr.2019.0442 32915693 PMC7482129

[B8] Congilosi J. L. Aga D. S. (2021). Review on the fate of antimicrobials, antimicrobial resistance genes, and other micropollutants in manure during enhanced anaerobic digestion and composting. J. Hazardous Materials 405, 123634. doi: 10.1016/j.jhazmat.2020.123634 33153790

[B9] Ding D. Wang B. Zhang X. Zhang J. Zhang H. Liu X.. (2023). The spread of antibiotic resistance to humans and potential protection strategies. Ecotoxicology Environ. Saf. 254, 114734. doi: 10.1016/j.ecoenv.2023.114734 36950985

[B10] European Biogas Association (2020). The ‘European Biomethane Map 2020’ shows a 51% increase of biomethane plants in Europe in two years.

[B11] eurostat Statistics Explained (2024). Agricultural production - livestock and meat.

[B12] Gao W. Li A. Ding G. Zhang K. Zhi S. (2022). Investigating changes in the characteristics of antibiotic resistance genes at different reaction stages of high solid anaerobic digestion with pig manure. Environ. pollut. 312, 120032. doi: 10.1016/j.envpol.2022.120032 36030955

[B13] German Federal Institute for Risk Assessment (2025). Antibiotic use in fattening animals: First annual report provides more precise data (No. 16/2023).

[B14] Gurmessa B. Ashworth A. J. Yang Y. Savin M. Moore P. A. Ricke S. C.. (2021). Variations in bacterial community structure and antimicrobial resistance gene abundance in cattle manure and poultry litter. Environ. Res. 197, 111011. doi: 10.1016/j.envres.2021.111011 33774017

[B15] Gutierrez C. Somoskovi A. Natarajan K. Bell D. (2018). Need for better adherence to optimal incubation temperature for quality laboratory diagnostics and antibiotic resistance monitoring. Afr J. Lab. Med. 7, 789. doi: 10.4102/ajlm.v7i2.789 30643735 PMC6325273

[B16] Jaleta M. Junker V. Kolte B. Börger M. Werner D. Dolsdorf C.. (2024). Improvements of weaned pigs barn hygiene to reduce the spread of antimicrobial resistance. Front. Microbiol. 15. doi: 10.3389/fmicb.2024.1393923 PMC11135127 38812683

[B17] Köninger J. Lugato E. Panagos P. Kochupillai M. Orgiazzi A. Briones M. J. I. (2021). Manure management and soil biodiversity: Towards more sustainable food systems in the EU. Agric. Syst. 194, 103251. doi: 10.1016/j.agsy.2021.103251

[B18] Li Y. Wang Q. Peng K. Liu Y. Li R. Wang Z. (2020). Emergence of carbapenem- and tigecycline-resistant proteus cibarius of animal origin. Front. Microbiol. 11. doi: 10.3389/fmicb.2020.01940 PMC7457074 32922378

[B19] Li L.-G. Yin X. Zhang T. (2018). Tracking antibiotic resistance gene pollution from different sources using machine-learning classification. Microbiome 6, 93. doi: 10.1186/s40168-018-0480-x 29793542 PMC5966912

[B20] Mortezaei Y. Williams M. R. Demirer G. N. (2024). The fate of antibiotic resistance genes during anaerobic digestion of sewage sludge with ultrasonic pretreatment. Environ. Sci. pollut. Res. 31, 5513–5525. doi: 10.1007/s11356-023-31558-6 38127236

[B21] Muurinen J. Stedtfeld R. Karkman A. Pärnänen K. Tiedje J. Virta M. (2017). Influence of manure application on the environmental resistome under finnish agricultural practice with restricted antibiotic use. Environ. Sci. Technol. 51, 5989–5999. doi: 10.1021/acs.est.7b00551 28453251

[B22] Okonkwo V. O. (2023). Antimicrobial resistance: molecular approaches to track antimicrobial resistance gene spread from decentralised septic tank wastewater (Scotland: University of Glasgow).

[B23] Partridge S. R. Kwong S. M. Firth N. Jensen S. O. (2018). Mobile genetic elements associated with antimicrobial resistance. Clin. Microbiol. Rev. 31. doi: 10.1128/CMR.00088-17 PMC6148190 30068738

[B24] Peng S. Zhang H. Song D. Chen H. Lin X. Wang Y.. (2022). Distribution of antibiotic, heavy metals and antibiotic resistance genes in livestock and poultry feces from different scale of farms in Ningxia, China. J. Hazardous Materials 440, 129719. doi: 10.1016/j.jhazmat.2022.129719 35985212

[B25] Prado J. Ribeiro H. Alvarenga P. Fangueiro D. (2022). A step towards the production of manure-based fertilizers: Disclosing the effects of animal species and slurry treatment on their nutrients content and availability. J. Cleaner Production 337, 130369. doi: 10.1016/j.jclepro.2022.130369

[B26] Riaz L. Wang Q. Yang Q. Li X. Yuan W. (2020). Potential of industrial composting and anaerobic digestion for the removal of antibiotics, antibiotic resistance genes and heavy metals from chicken manure. Sci. Total Environ. 718, 137414. doi: 10.1016/j.scitotenv.2020.137414 32105920

[B27] Skandalis N. Maeusli M. Papafotis D. Miller S. Lee B. Theologidis I.. (2021). Environmental spread of antibiotic resistance. Antibiotics (Basel) 10. doi: 10.3390/antibiotics10060640 PMC8226744 34071771

[B28] Song W. Wang X. Gu J. Zhang S. Yin Y. Li Y.. (2017). Effects of different swine manure to wheat straw ratios on antibiotic resistance genes and the microbial community structure during anaerobic digestion. Bioresource Technol. 231, 1–8. doi: 10.1016/j.biortech.2017.01.054 28171769

[B29] Stedtfeld R. D. Guo X. Stedtfeld T. M. Sheng H. Williams M. R. Hauschild K.. (2018). Primer set 2.0 for highly parallel qPCR array targeting antibiotic resistance genes and mobile genetic elements. FEMS Microbiol. Ecol. 94. doi: 10.1093/femsec/fiy130 PMC7250373 30052926

[B30] Sun B. Bai Z. Li R. Song M. Zhang J. Wang J.. (2024). Efficient elimination of antibiotic resistome in livestock manure by semi-permeable membrane covered hyperthermophilic composting. Bioresource Technol. 407, 131134. doi: 10.1016/j.biortech.2024.131134 39038713

[B31] Tada T. Miyoshi-Akiyama T. Shimada K. Shiroma A. Nakano K. Teruya K.. (2016). A Carbapenem-Resistant Pseudomonas aeruginosa Isolate Harboring Two Copies of blaIMP-34 Encoding a Metallo-β-Lactamase. PloS One 11, e0149385. doi: 10.1371/journal.pone.0149385 27055243 PMC4824433

[B32] Thomas C. (2023). Manure management measures to reduce the risk of spreading ESBL-/AmpCproducing Escherichia coli from chicken manure into the food chain (Leibniz-Institut für Agrartechnik und Bioökonomie: Freie Universität Berlin).

[B33] Wang L. Wang J. Wang J. Zhu L. Yang L. Yang R. (2019). Distribution characteristics of antibiotic resistant bacteria and genes in fresh and composted manures of livestock farms. Sci. Total Environ. 695, 133781. doi: 10.1016/j.scitotenv.2019.133781 31756854

[B34] Waseem H. Jameel S. Ali J. Saleem Ur Rehman H. Tauseef I. Farooq U.. (2019). Contributions and challenges of high throughput qPCR for determining antimicrobial resistance in the environment: A critical review. Molecules 24. doi: 10.3390/molecules24010163 PMC6337382 30609875

[B35] World Health Organization (2023). Antimicrobial resistance.

[B36] World Health Organization (2024). “A risk management tool for mitigating antimicrobial resistance due to non-human use (No. ISBN 978-92-4-008461-2),” in WHO List of Medically Important Antimicrobials: a risk management tool for mitigating antimicrobial resistance due to non-human use. (Geneva: World Health Organization)

[B37] Yangin-Gomec C. Sapmaz T. Aydin S. (2020). Impact of inoculum acclimation on energy recovery and investigation of microbial community changes during anaerobic digestion of the chicken manure. Environ. Technol. 41, 49–58. doi: 10.1080/09593330.2018.1551434 30461343

[B38] Yin Y. He X. Szewczyk P. Nguyen T. Chang G. (2006). Structure of the multidrug transporter EmrD from Escherichia coli. Science 312, 741–744. doi: 10.1126/science.1125629 16675700 PMC3152482

[B39] Yue Z. Zhang J. Zhou Z. Ding C. Zhang T. Wan L.. (2022). Antibiotic degradation dominates the removal of antibiotic resistance genes during composting. Bioresource Technol. 344, 126229. doi: 10.1016/j.biortech.2021.126229 34737135

[B40] Zahedi S. Gros M. Casabella O. Petrovic M. Balcazar J. L. Pijuan M. (2022). Occurrence of veterinary drugs and resistance genes during anaerobic digestion of poultry and cattle manures. Sci. Total Environ. 822, 153477. doi: 10.1016/j.scitotenv.2022.153477 35093343

[B41] Zhang Y. Banks C. J. Heaven S. (2012). Co-digestion of source segregated domestic food waste to improve process stability. Bioresource Technol. 114, 168–178. doi: 10.1016/j.biortech.2012.03.040 22472639

[B42] Zhang J. Lu T. Shen P. Sui Q. Zhong H. Liu J.. (2019). The role of substrate types and substrate microbial community on the fate of antibiotic resistance genes during anaerobic digestion. Chemosphere 229, 461–470. doi: 10.1016/j.chemosphere.2019.05.036 31091487

[B43] Zhang C. Xiao G. Peng L. Su H. Tan T. (2013). The anaerobic co-digestion of food waste and cattle manure. Bioresource Technol. 129, 170–176. doi: 10.1016/j.biortech.2012.10.138 23246757

[B44] Zhuang M. Achmon Y. Cao Y. Liang X. Chen L. Wang H.. (2021). Distribution of antibiotic resistance genes in the environment. Environ. pollut. 285, 117402. doi: 10.1016/j.envpol.2021.117402 34051569

