# Supplementary figures and images for: Temporal dynamics of antimicrobial resistance gene abundances in chicken manure and anaerobic digestate
Source: Front Antibiot. 2025 Jun 27;4:1612886. doi: 10.3389/frabi.2025.1612886 (PMC12245806; doi:10.3389/frabi.2025.1612886)

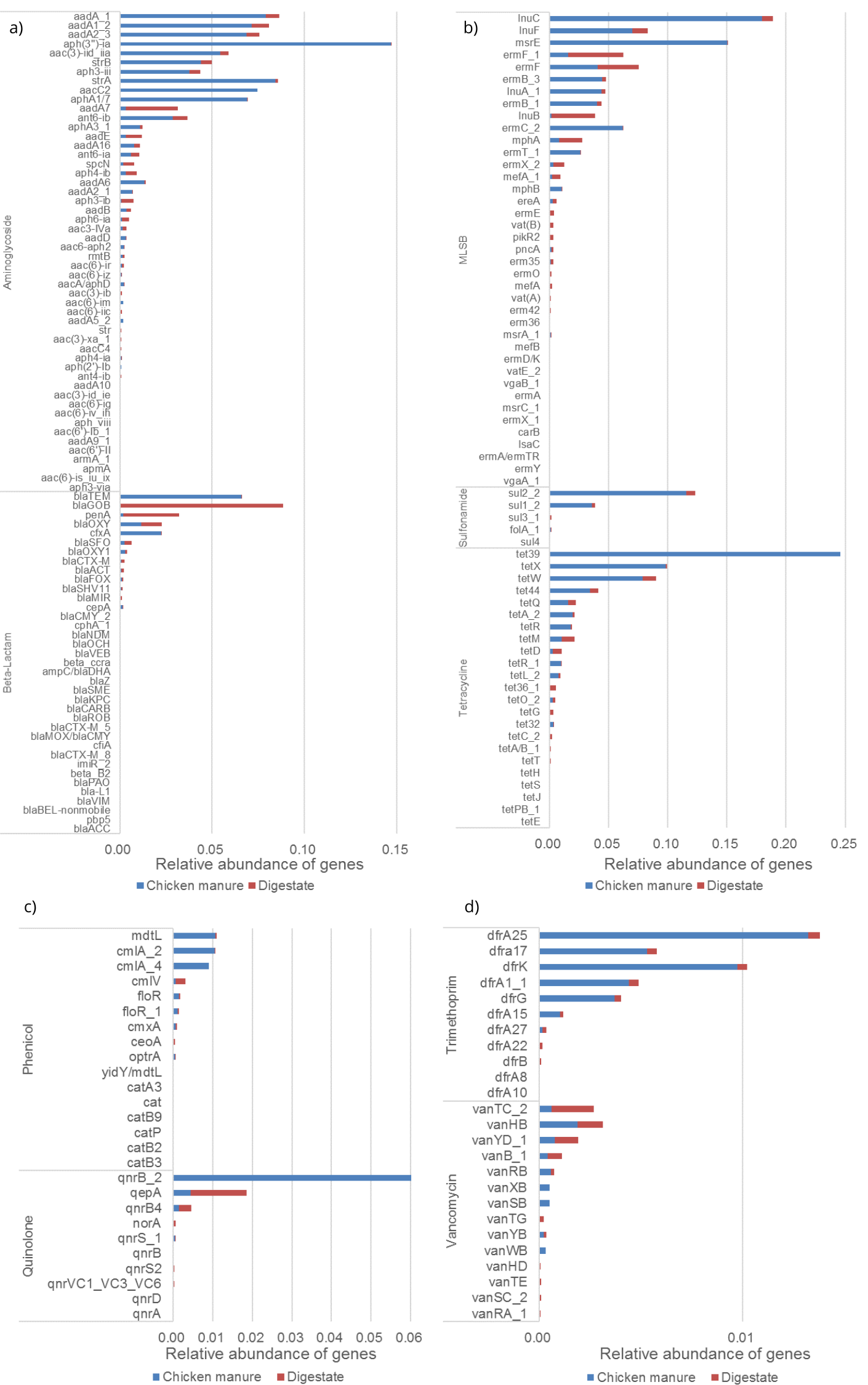

Supplement: Supplementary file 1 [file Image1.png]

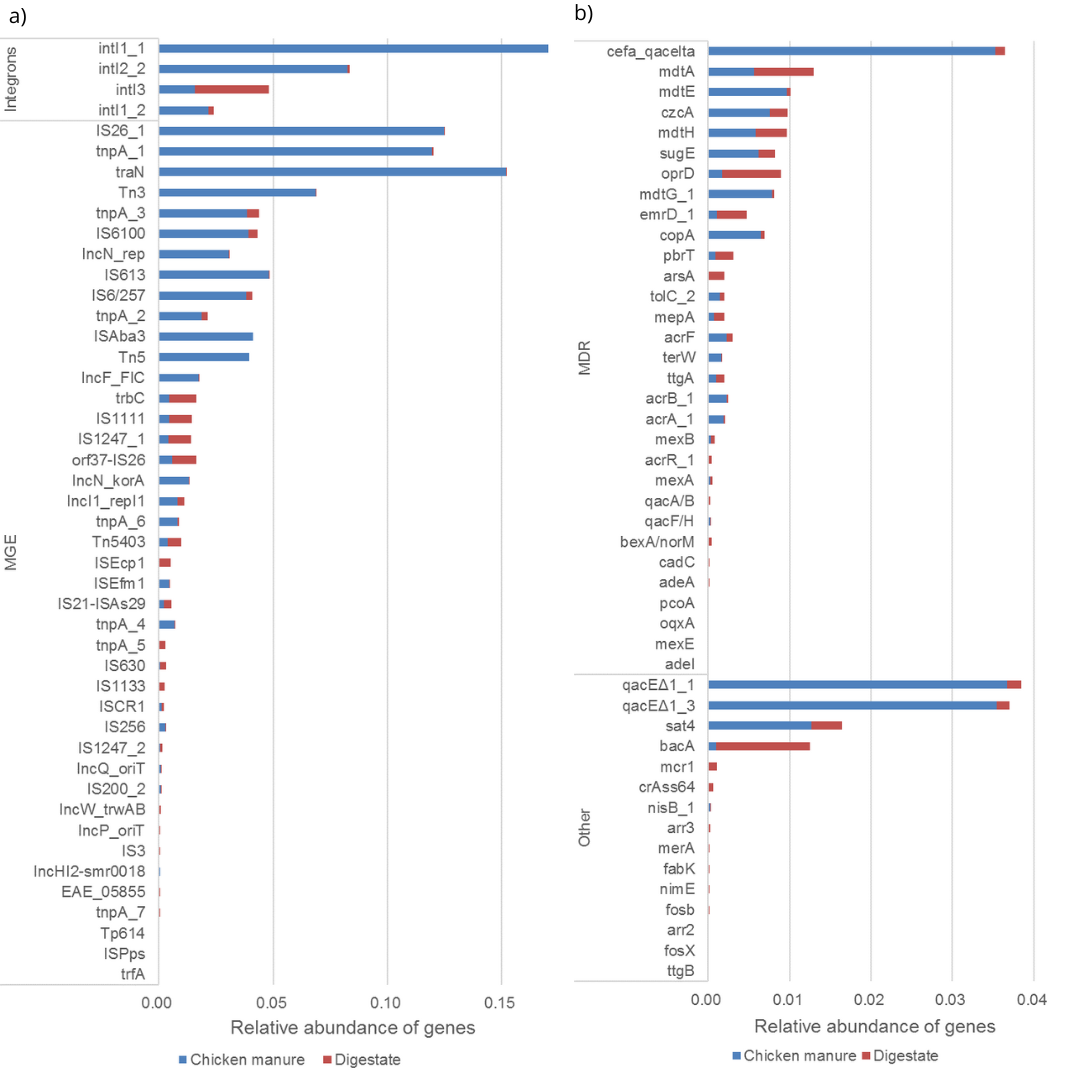

Supplement: Supplementary file 2 [file Image2.png]

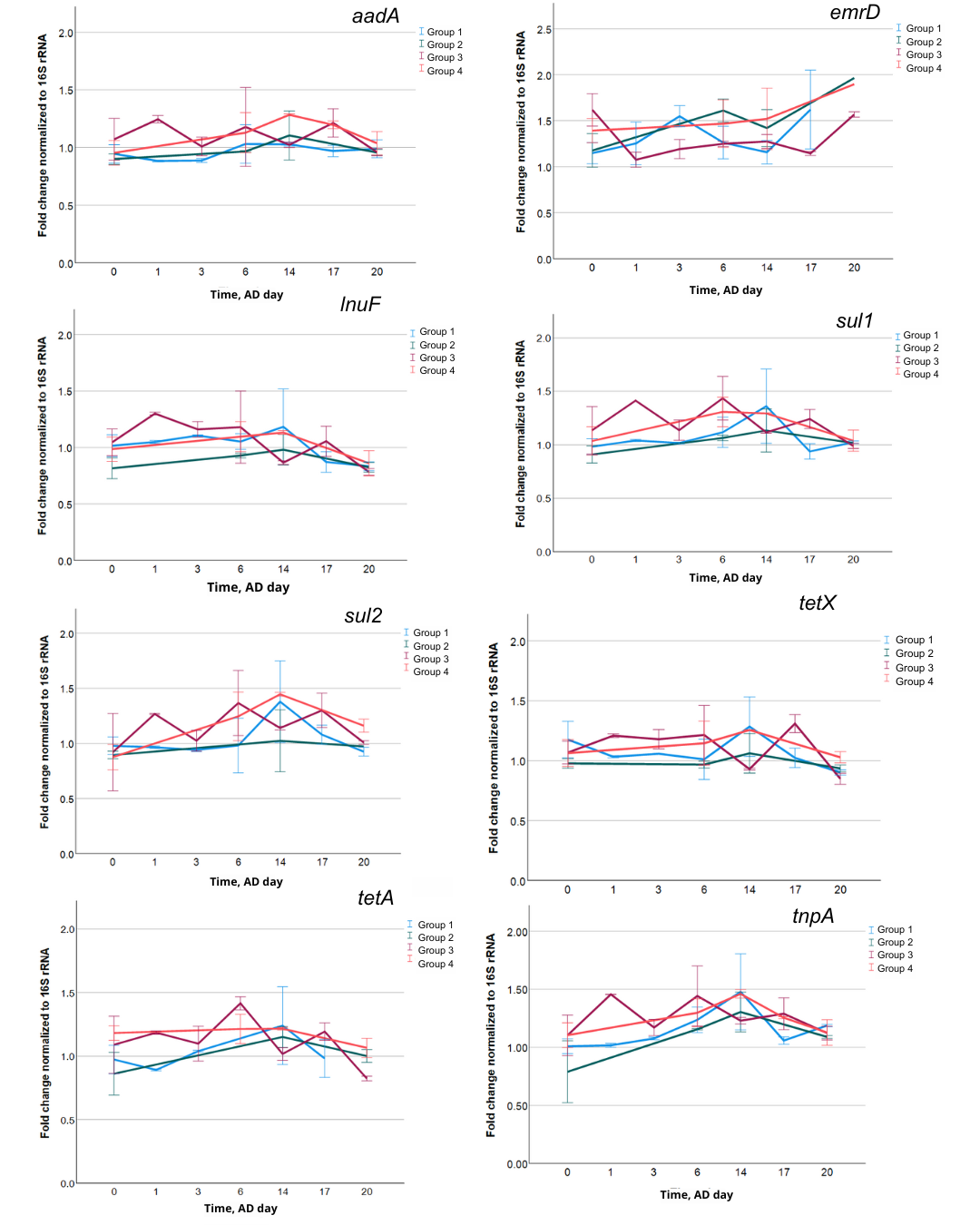

Supplement: Supplementary file 3 [file Image3.png]
