# Supplementary material for: Temporal dynamics of antimicrobial resistance gene abundances in chicken manure and anaerobic digestate
Source: Front Antibiot. 2025 Jun 27;4:1612886. doi: 10.3389/frabi.2025.1612886 (PMC12245806; doi:10.3389/frabi.2025.1612886)
Supplement: Supplementary file 4 [file Table1.docx]

Supplementary Material

# Supplementary Figures

Figure 1 Antibiotic resistance genes (ARGs) relative abundance normalized to 16S rRNA on Resistomap HT-qPCR SmartChip in chicken manure and digestate. a) ARGs from class Aminoglycoside and Beta-lactam; b) Macrolide-Lincosamide-Streptogramin B (MLSB), Sulfomanide, Tetracycline; c) Phenicol and Quinolone; d) Trimethroprim and Vancomycin.

Figure 2 Antibiotic resistance genes (ARGs) relative abundance normalized to 16S rRNA on Resistomap HT-qPCR SmartChip in chicken manure and digestate. a) ARGs from class Integrons and Mobile Genetic Elements (MGE). Relative abundance for *intl1_1* was 1.73 (not shown) b) Multdrugs Resistance (MDR) and Other.

Figure 3 Average fold change of selected ARGs normalized to 16S rRNA in digestate across all selected time points and all setups. Group 1: AD at 30°C with chicken manure; Group 2: AD at 30°C with chicken manure and sawdust; Group 3: AD at 37°C with chicken manure; Group 4: AD at 37°C with chicken manure and sawdust. Each sample was measured in triplicates. Error bars represent the standard error.

# Supplementary Tables

Table 1 Statistical analysis from UNIANOVA model for chicken manure samples considering different age and flocks.

Table 2 Statistical analysis from UNIANOVA model for digestate samples, considering different time points, temperature (temp) and sawdust (substr).

Table 1 Statistical analysis from UNIANOVA model for chicken manure samples considering different age and flocks.

| Gene | Parameter | Regression coefficient | *P* value | 95% Confidence Interval | |
| --- | --- | --- | --- | --- | --- |
|  |  |  |  | Lower Bound | Upper Bound |
| *aadA* | Intercept | 3.660 | <0.001 | 3.406 | 3.914 |
|  | [age=1] | -2.098 | <0.001 | -2.500 | -1.697 |
|  | [age=3] | -1.810 | <0.001 | -2.318 | -1.302 |
|  | [age=5] |  | <0.001 |  |  |
|  | [flock=4] | -0.493 | 0.057 | -1.001 | 0.014 |
|  | [flock=5] | -0.713 | 0.026 | -1.335 | -0.091 |
|  | [flock=6] | -1.687 | <0.001 | -2.194 | -1.179 |
|  | [flock=10] | 2.843 | <0.001 | 2.221 | 3.465 |
|  | [flock=11] |  | <0.001 |  |  |
|  | [age=1] * [flock=4] | 0.332 | 0.371 | -0.409 | 1.072 |
|  | [age=1] * [flock=5] | 0.320 | 0.401 | -0.442 | 1.082 |
|  | [age=1] * [flock=6] | 1.087 | 0.002 | 0.415 | 1.758 |
|  | [age=1] * [flock=10] | -3.562 | <0.001 | -4.384 | -2.739 |
|  | [age=1] * [flock=11] |  | <0.001 |  |  |
|  | [age=3] * [flock=4] | 1.533 | <0.001 | 0.730 | 2.336 |
|  | [age=3] * [flock=6] | 2.687 | <0.001 | 1.884 | 3.490 |
| *emrD* | Intercept | 6.090 | <0.001 | 4.510 | 7.67 |
|  | [age=1] | -4.570 | <0.001 | -6.804 | -2.336 |
|  | [age=3] | -1.603 | 0.152 | -3.838 | 0.631 |
|  | [age=5] |  | 0.023 |  |  |
|  | [flock=4] | -2.637 | 0.023 | -4.871 | -0.402 |
|  | [flock=5] | -1.567 | 0.161 | -3.801 | 0.668 |
|  | [flock=6] | -1.133 | 0.307 | -3.368 | 1.101 |
|  | [flock=10] | 1.043 | 0.346 | -1.191 | 3.278 |
|  | [flock=11] |  | 0.117 |  |  |
|  | [age=1] * [flock=4] | 7.020 | <0.001 | 3.860 | 10.180 |
|  | [age=1] * [flock=5] | 3.995 | 0.011 | 0.997 | 6.992 |
|  | [age=1] * [flock=6] | 1.087 | 0.486 | -2.073 | 4.247 |
|  | [age=1] * [flock=10] | -1.050 | 0.501 | -4.210 | 2.110 |
|  | [age=1] * [flock=11] |  | <0.001 |  |  |
|  | [age=3] * [flock=4] | 2.920 | 0.069 | -0.240 | 6.080 |
| *lnuF* | Intercept | 1.227 | <0.001 | 0.979 | 1.474 |
|  | [age=1] | -0.238 | 0.176 | -0.588 | 0.111 |
|  | [age=3] | -0.347 | 0.110 | -0.775 | 0.082 |
|  | [age=5] |  | 0.240 |  |  |
|  | [flock=4] | -0.470 | 0.032 | -0.898 | -0.042 |
|  | [flock=5] | 0.347 | 0.164 | -0.148 | 0.841 |
|  | [flock=6] | -0.407 | 0.062 | -0.835 | 0.022 |
|  | [flock=10] | -0.343 | 0.168 | -0.838 | 0.151 |
|  | [flock=11] |  | 0.777 |  |  |
|  | [age=1] * [flock=4] | 0.065 | 0.829 | -0.541 | 0.671 |
|  | [age=1] * [flock=5] | -0.513 | 0.094 | -1.119 | 0.093 |
|  | [age=1] * [flock=6] | 0.122 | 0.659 | -0.431 | 0.675 |
|  | [age=1] * [flock=10] | 0.828 | 0.014 | 0.174 | 1.483 |
|  | [age=1] * [flock=11] |  | <0.001 |  |  |
|  | [age=3] * [flock=4] | 0.860 | 0.011 | 0.206 | 1.514 |
|  | [age=3] * [flock=6] | 1.033 | 0.003 | 0.379 | 1.688 |
| *sul1* | Intercept | 2.413 | <0.001 | 2.256 | 2.569 |
|  | [age=1] | -0.616 | <0.001 | -0.879 | -0.353 |
|  | [age=3] | -2.053 | <0.001 | -2.391 | -1.715 |
|  | [age=5] |  | <0.001 |  |  |
|  | [flock=4] | -0.796 | <0.001 | -1.134 | -0.458 |
|  | [flock=5] | 0.993 | <0.001 | 0.570 | 1.417 |
|  | [flock=6] | -0.939 | <0.001 | -1.277 | -0.602 |
|  | [flock=10] | 2.317 | <0.001 | 1.893 | 2.740 |
|  | [flock=11] |  | <0.001 |  |  |
|  | [age=1] * [flock=4] | -0.067 | 0.787 | -0.566 | 0.431 |
|  | [age=1] * [flock=5] | -1.637 | <0.001 | -2.155 | -1.118 |
|  | [age=1] * [flock=6] | 0.078 | 0.730 | -0.374 | 0.529 |
|  | [age=1] * [flock=10] | -2.420 | <0.001 | -2.980 | -1.860 |
|  | [age=3] * [flock=4] | 2.636 | <0.001 | 2.094 | 3.178 |
|  | [age=3] * [flock=6] | 2.796 | <0.001 | 2.254 | 3.338 |
| *sul2* | Intercept | 3.005 | <0.001 | 2.865 | 3.146 |
|  | [age=1] | -1.607 | <0.001 | -1.844 | -1.370 |
|  | [age=3] | -2.439 | <0.001 | -2.743 | -2.134 |
|  | [age=5] |  | <0.001 |  |  |
|  | [flock=4] | -0.872 | <0.001 | -1.109 | -0.635 |
|  | [flock=5] | 1.480 | <0.001 | 1.098 | 1.862 |
|  | [flock=6] | -1.149 | <0.001 | -1.453 | -0.844 |
|  | [flock=10] | 1.933 | <0.001 | 1.552 | 2.315 |
|  | [flock=11] |  | <0.001 |  |  |
|  | [age=1] * [flock=4] | 1.767 | <0.001 | 1.36 | 2.174 |
|  | [age=1] * [flock=5] | -2.002 | <0.001 | -2.469 | -1.534 |
|  | [age=1] * [flock=6] | 0.380 | 0.066 | -0.026 | 0.787 |
|  | [age=1] * [flock=10] | -1.918 | <0.001 | -2.423 | -1.413 |
|  | [age=1] * [flock=11] |  | <0.001 |  |  |
|  | [age=3] * [flock=4] | 2.865 | <0.001 | 2.416 | 3.315 |
|  | [age=3] * [flock=6] | 2.442 | <0.001 | 1.954 | 2.930 |
| *tetA* | Intercept | 3.223 | <0.001 | 2.977 | 3.470 |
|  | [age=1] | -1.363 | <0.001 | -1.790 | -0.937 |
|  | [age=3] | -1.973 | <0.001 | -2.400 | -1.547 |
|  | [age=5] |  | <0.001 |  |  |
|  | [flock=4] | -0.890 | <0.001 | -1.317 | -0.463 |
|  | [flock=5] | 1.303 | <0.001 | 0.811 | 1.796 |
|  | [flock=6] | 0.803 | 0.002 | 0.311 | 1.296 |
|  | [flock=10] | 1.380 | <0.001 | 0.887 | 1.873 |
|  | [flock=11] |  | <.001 |  |  |
|  | [age=1] * [flock=5] | -0.923 | 0.007 | -1.575 | -0.271 |
|  | [age=1] * [flock=6] | -1.652 | <0.001 | -2.304 | -1.000 |
|  | [age=1] * [flock=10] | -1.743 | <0.001 | -2.440 | -1.046 |
|  | [age=1] * [flock=11] |  | <0.001 |  |  |
|  | [age=3] * [flock=4] | 2.227 | <0.001 | 1.575 | 2.879 |
| *tetX* | Intercept | 0.767 | <0.001 | 0.555 | 0.978 |
|  | [age=1] | 0.328 | 0.054 | -0.006 | 0.663 |
|  | [age=3] | -0.197 | 0.353 | -0.620 | 0.226 |
|  | [age=5] |  | 0.402 |  |  |
|  | [flock=4] | -0.083 | 0.693 | -0.506 | 0.34 |
|  | [flock=5] | 0.177 | 0.495 | -0.341 | 0.695 |
|  | [flock=6] | 0.650 | 0.003 | 0.227 | 1.073 |
|  | [flock=10] | 0.143 | 0.579 | -0.375 | 0.661 |
|  | [flock=11] |  | 0.302 |  |  |
|  | [age=1] * [flock=4] | -0.155 | 0.614 | -0.772 | 0.462 |
|  | [age=1] * [flock=5] | -0.760 | 0.020 | -1.395 | -0.125 |
|  | [age=1] * [flock=6] | -1.232 | <0.001 | -1.791 | -0.672 |
|  | [age=1] * [flock=10] | -0.605 | 0.082 | -1.290 | 0.080 |
|  | [age=1] * [flock=11] |  | 0.002 |  |  |
|  | [age=3] * [flock=4] | 0.397 | 0.238 | -0.272 | 1.066 |
|  | [age=3] * [flock=6] | -0.207 | 0.536 | -0.876 | 0.462 |
| *tnpA* | Intercept | 1.194 | <0.001 | 1.051 | 1.336 |
|  | [age=1] | 0.073 | 0.502 | -0.145 | 0.291 |
|  | [age=3] | -0.280 | 0.044 | -0.553 | -0.007 |
|  | [age=5] |  | <0.001 |  |  |
|  | [flock=4] | -0.437 | 0.002 | -0.710 | -0.164 |
|  | [flock=5] | 0.427 | 0.012 | 0.097 | 0.756 |
|  | [flock=6] | -0.504 | <0.001 | -0.777 | -0.231 |
|  | [flock=10] | 0.670 | <0.001 | 0.341 | 0.999 |
|  | [flock=11] |  | <0.001 |  |  |
|  | [age=1] * [flock=4] | -0.073 | 0.711 | -0.468 | 0.322 |
|  | [age=1] * [flock=5] | -0.355 | 0.083 | -0.758 | 0.048 |
|  | [age=1] * [flock=6] | 0.019 | 0.916 | -0.340 | 0.378 |
|  | [age=1] * [flock=10] | -1.013 | <0.001 | -1.449 | -0.578 |
|  | [age=1] * [flock=11] |  | <0.001 |  |  |
|  | [age=3] * [flock=4] | 0.757 | <0.001 | 0.329 | 1.185 |
|  | [age=3] * [flock=6] | 0.890 | <0.001 | 0.463 | 1.318 |

Table 2 Statistical analysis from UNIANOVA model for digestate samples, considering different time points, temperature (temp) and sawdust (substr).

| Gene | Parameter | Regression cofficient | *P* value | 95% Confidence Interval | |
| --- | --- | --- | --- | --- | --- |
|  |  |  |  | Lower Bound | Upper Bound |
| *aadA* | Intercept | 0.994 | <0.001 | 0.874 | 1.114 |
|  | [day=0] | -0.004 | 0.951 | -0.127 | 0.119 |
|  | [day=1] | 0.051 | 0.598 | -0.139 | 0.240 |
|  | [day=3] | -0.036 | 0.668 | -0.200 | 0.129 |
|  | [day=6] | 0.113 | 0.107 | -0.025 | 0.251 |
|  | [day=14] | 0.242 | 0.001 | 0.100 | 0.384 |
|  | [day=17] | 0.102 | 0.204 | -0.056 | 0.259 |
|  | [day=20] |  | <0.001 |  |  |
|  | [temp=30] | -0.095 | 0.008 | -0.165 | -0.025 |
|  | [substr=0] | 0.040 | 0.299 | -0.036 | 0.116 |
| *lnuF* | Intercept | 0.798 | <0.001 | 0.694 | 0.902 |
|  | [day=0] | 0.169 | 0.002 | 0.062 | 0.275 |
|  | [day=1] | 0.347 | <0.001 | 0.169 | 0.524 |
|  | [day=3] | 0.302 | <0.001 | 0.154 | 0.449 |
|  | [day=6] | 0.276 | <0.001 | 0.153 | 0.399 |
|  | [day=14] | 0.256 | <0.001 | 0.134 | 0.378 |
|  | [day=17] | 0.111 | 0.140 | -0.037 | 0.260 |
|  | [day=20] |  | <0.001 |  |  |
|  | [temp=30] | -0.070 | 0.036 | -0.136 | -0.005 |
|  | [substr=0] | 0.065 | 0.080 | -0.008 | 0.138 |
| *sul1* | Intercept | 1.006 | <0.001 | 0.883 | 1.128 |
|  | [day=0] | 0.043 | 0.495 | -0.082 | 0.169 |
|  | [day=1] | 0.187 | 0.101 | -0.037 | 0.411 |
|  | [day=3] | 0.076 | 0.370 | -0.092 | 0.244 |
|  | [day=6] | 0.271 | <0.001 | 0.131 | 0.411 |
|  | [day=14] | 0.269 | <0.001 | 0.127 | 0.411 |
|  | [day=20] |  | <0.001 |  |  |
|  | [day=17] | 0.053 | 0.536 | -0.117 | 0.223 |
|  | [temp=30] | -0.132 | <0.001 | -0.207 | -0.058 |
|  | [substr=0] | 0.060 | 0.151 | -0.022 | 0.143 |
| *sul2* | Intercept | 0.913 | <0.001 | 0.739 | 1.088 |
|  | [day=0] | 0.016 | 0.858 | -0.161 | 0.193 |
|  | [day=1] | 0.158 | 0.251 | -0.114 | 0.430 |
|  | [day=3] | 0.038 | 0.751 | -0.198 | 0.274 |
|  | [day=6] | 0.250 | 0.017 | 0.045 | 0.456 |
|  | [day=14] | 0.350 | <0.001 | 0.146 | 0.553 |
|  | [day=17] | 0.228 | 0.070 | -0.019 | 0.475 |
|  | [day=20] |  | <0.001 |  |  |
|  | [temp=30] | -0.121 | 0.025 | -0.226 | -0.015 |
|  | [substr=0] | 0.087 | 0.148 | -0.031 | 0.205 |
| *tetA* | Intercept | 1.003 | <0.001 | 0.861 | 1.145 |
|  | [day=0] | 0.074 | 0.341 | -0.081 | 0.229 |
|  | [day=1] | 0.079 | 0.458 | -0.133 | 0.291 |
|  | [day=3] | 0.133 | 0.214 | -0.079 | 0.346 |
|  | [day=6] | 0.280 | 0.005 | 0.087 | 0.472 |
|  | [day=14] | 0.208 | 0.019 | 0.035 | 0.381 |
|  | [day=17] | 0.130 | 0.208 | -0.075 | 0.335 |
|  | [day=20] |  | <0.001 |  |  |
|  | [temp=30] | -0.115 | 0.015 | -0.207 | -0.023 |
|  | [substr=0] | -0.005 | 0.923 | -0.107 | 0.097 |
| *tnpA* | Intercept | 1.149 | <0.001 | 1.004 | 1.293 |
|  | [day=0] | -0.106 | 0.163 | -0.255 | 0.044 |
|  | [day=1] | 0.065 | 0.575 | -0.164 | 0.294 |
|  | [day=3] | -0.029 | 0.770 | -0.228 | 0.170 |
|  | [day=6] | 0.178 | 0.034 | 0.014 | 0.343 |
|  | [day=14] | 0.298 | 0.001 | 0.123 | 0.473 |
|  | [day=17] | 0.035 | 0.714 | -0.155 | 0.226 |
|  | [day=20] |  | <0.001 |  |  |
|  | [temp=30] | -0.163 | <0.001 | -0.248 | -0.078 |
|  | [substr=0] | 0.076 | 0.106 | -0.017 | 0.169 |
| *emrD* | Intercept | 1.896 | <0.001 | 1.467 | 2.325 |
|  | [day=0] | -0.458 | 0.045 | -0.905 | -0.012 |
|  | [day=1] | -0.491 | 0.010 | -0.863 | -0.119 |
|  | [day=3] | -0.375 | 0.011 | -0.663 | -0.087 |
|  | [day=6] | -0.399 | 0.094 | -0.867 | 0.070 |
|  | [day=14] | -0.371 | 0.125 | -0.848 | 0.106 |
|  | [day=17] | -0.423 | 0.026 | -0.794 | -0.051 |
|  | [day=20] |  | <0.001 |  |  |
|  | [temp=30] | 0.067 | 0.825 | -0.540 | 0.675 |
|  | [substr=0] | -0.329 | 0.175 | -0.809 | 0.151 |
|  | [day=0] * [substr=0] | 0.418 | 0.104 | -0.088 | 0.924 |
|  | [day=6] * [substr=0] | 0.051 | 0.849 | -0.478 | 0.580 |
|  | [day=14] * [substr=0] | 0.073 | 0.783 | -0.456 | 0.602 |
|  | [day=20] * [substr=0] |  | 0.016 |  |  |
|  | [day=0] * [temp=30] | -0.381 | 0.229 | -1.008 | 0.246 |
|  | [day=1] * [temp=30] | 0.110 | 0.763 | -0.613 | 0.832 |
|  | [day=3] * [temp=30] | 0.290 | 0.400 | -0.393 | 0.973 |
|  | [day=6] * [temp=30] | 0.005 | 0.987 | -0.641 | 0.652 |
|  | [day=14] * [temp=30] | -0.177 | 0.589 | -0.825 | 0.472 |
|  | [day=17] * [temp=30] | 0.410 | 0.251 | -0.296 | 1.115 |
|  | [day=20] * [temp=30] |  | <0.001 |  |  |
| *tetX* | Intercept | 0.952 | <0.001 | 0.809 | 1.096 |
|  | [day=0] | 0.138 | 0.067 | -0.010 | 0.286 |
|  | [day=1] | 0.323 | 0.013 | 0.069 | 0.576 |
|  | [day=3] | 0.293 | 0.003 | 0.103 | 0.482 |
|  | [day=6] | 0.270 | 0.002 | 0.099 | 0.441 |
|  | [day=14] | 0.173 | 0.057 | -0.005 | 0.352 |
|  | [day=17] | 0.423 | 0.001 | 0.169 | 0.676 |
|  | [day=20] |  | 0.002 |  |  |
|  | [substr=0] | 0.067 | 0.203 | -0.037 | 0.170 |
|  | [temp=30] | -0.110 | 0.317 | -0.327 | 0.107 |
|  | [day=0] * [temp=30] | 0.020 | 0.864 | -0.209 | 0.248 |
|  | [day=1] * [temp=30] | -0.284 | 0.110 | -0.634 | 0.066 |
|  | [day=3] * [temp=30] | -0.229 | 0.172 | -0.561 | 0.102 |
|  | [day=6] * [temp=30] | -0.217 | 0.104 | -0.480 | 0.046 |
|  | [day=14] * [temp=30] | 0.085 | 0.511 | -0.172 | 0.342 |
|  | [day=17] * [temp=30] | -0.393 | 0.023 | -0.732 | -0.055 |
|  | [day=20] * [temp=30] |  | 0.015 |  |  |
|  | [temp=30] * [substr=0] | 0.218 | 0.005 | 0.068 | 0.368 |
